# Mental health care access among Latinx and Indigenous Mexican immigrants in rural desert communities in the COVID-19 pandemic: a community-engaged study

**DOI:** 10.1186/s12889-026-27551-6

**Published:** 2026-05-04

**Authors:** Noah Baltrushes, James Elkins, Stephanie Martinez, Ann Marie Cheney

**Affiliations:** Department of Social Medicine, Population and Public Health, University of California Riverside School of Medicine, SOM Bldg 1, SMPPH, 900 University Avenue, Riverside, CA 92521 USA

**Keywords:** Latinx immigrant health, Mental health, Indigenous Mexicans, Barriers to healthcare access, COVID-19 pandemic, Community-based participatory research

## Abstract

**Background:**

Structural and social inequities greatly impacted the mental and physical health of Latinx and Indigenous Mexican populations Hispanic/Latino immigrants in rural communities in the COVID-19 pandemic. These populations experienced disproportionate infection and mortality rates, exacerbated by existing structural and social inequities, creating unique stressors that negatively impacted community members’ mental health. Limited financial resources, lack of healthcare infrastructure, fear of deportation, and stigma shaped decisions to access mental healthcare services. The historical economic instability, social isolation, and limited healthcare access experienced by this group set the stage for the challenges they faced throughout the pandemic.

**Methods:**

Between September 2022 and June 2024, qualitative, semi-structured interviews were conducted with 12 participants, focusing on their mental health experiences during the pandemic. The socioecological model was used as a conceptual framework to contextualize findings with multiple spheres of influence.

**Results:**

At the macro level, structural inequities such as precarious labor, exclusionary immigration policies, and underinvestment in rural regions, set the stage for pandemic-related stressors such as isolation, loss, and misinformation. At the individual level, fear of deportation and stigma surrounding mental health discouraged help-seeking behaviors. These intersecting influences underscore the socioecological determinants that shaped mental health care access among rural Latinx and Indigenous Mexican communities.

**Conclusions:**

Structural interventions are needed to reduce inequities, rebuild trust, and improve access to mental healthcare services in underserved, rural populations.

**Supplementary Information:**

The online version contains supplementary material available at 10.1186/s12889-026-27551-6.

## Introduction

The COVID-19 pandemic revealed and exacerbated health disparities in underserved communities across the United States (US), particularly among Latinx and Indigenous populations [1]. These groups faced increased COVID-19 infection rates [2], with Latinx individuals comprising 38.9% of California’s population but representing 47.1% of cases in the first year of the pandemic [3]. Indigenous populations experienced COVID-19 mortality rates twice that of White populations [4].

Marginalized communities also suffered disproportionately from the mental health impacts of the pandemic, including heightened rates of depression, anxiety, and substance abuse [5]. In rural areas, predominantly inhabited by Latinx agricultural workers, economic instability, social isolation, and strained access to healthcare compounded mental health challenges, further disrupting personal relationships, employment, and family dynamics [5]. These structural and social inequities perpetuated a cycle of disadvantage, increasing the risk of COVID-19 infection and mental health disparities [6]. Research on mental health among rural Hispanic/Latino populations during the pandemic indicates additional burden due to the isolating conditions, economic inequities, and fear of contracting COVID-19, as well as heightened anxiety and depression [7, 8]. The Hispanic/Latino population is the largest racial/ethnic group in rural America, and many work as farm laborers in agrobusiness [9, 10]. Addressing systemic and individual unmet mental health needs and preparing for future public health responses in this rural minority population requires understanding the structural and social inequities that underpin mental health burdens among Latinx and Indigenous Mexican immigrants in rural farm working communities.

While prior research has documented the disproportionate mental health impacts of COVID-19 among Latinx and Indigenous populations and the role of structural inequities in shaping these outcomes [1, 3, 6–10], fewer studies have examined how immigration-related fear, Indigenous identity, rural geographic isolation, and reliance on safety-net free clinics intersect to shape mental health trajectories over time. This study addresses this gap by providing in-depth, community-engaged qualitative data from Latinx and Indigenous Mexican immigrants living in a rural desert region of Inland Southern California. Using a socioecological framework [11], we examine how macro-level immigration policy and structural underinvestment interact with meso-level service constraints and individual experiences of anxiety, depression, and grief across pre-pandemic, pandemic, and post-pandemic periods.

## Methods

### Study design and objectives

This study, carried out from September 2022 to June 2024, employed a Community-Based Participatory Research (CBPR) approach to investigate perceptions and experiences of Latinx and Indigenous Mexican immigrant communities during the COVID-19 pandemic in a nuanced and culturally competent manner. CBPR emphasizes a collaborative partnership between researchers and community members, promoting co-learning, capacity building, and long-term engagement [12], and effectively addresses health inequities and fosters community-driven solutions [13]. The idea for this study emerged from ongoing, federally funded research conducted through Unidas por Salud, a long-standing community-academic partnership that brings together community health workers (CHWs)/promotoras, students, and local community and healthcare system partners in the region. Prior engagement activities and research carried out by the community-academic partnership had identified unmet mental health needs among Latinx and Indigenous community members during the pandemic [14]. In response, the team secured additional funding to better understand how these needs were being addressed by existing healthcare services.

In alignment with CBPR principles, community and academic partners co-designed the study, including the consent process, recruitment strategy, and data collection, to identify the role of the pandemic in exacerbating existing mental health needs and barriers to healthcare access. The community-academic team that led this research included CHWs trained in human subjects research who played an active role in engaging participants in study activity. As members of the local community, CHWs provided linguistically and culturally concordant recruitment and study engagement, leveraging established trust, local knowledge, and experience navigating health and social service systems.

This study was reviewed and approved by the Institutional Review Board (IRB) of the University of California, Riverside. All participants provided informed consent prior to participation, and the study adhered to the ethical principles outlined in the Declaration of Helsinki.

### Setting

Our study focused on the influence of the pandemic on mental health burden among a patient population accessing primary care services at a free clinic offering once-a-month Saturday services in the Eastern Coachella Valley, a rural desert region in Inland Southern California [12]. The clinic provides co-located mental healthcare services using a brief intervention model consisting of a 30-minute consultation and referral for follow up care. The clinic provides services regardless of insurance status along with access to linguistically appropriate services, including Spanish and Purépecha, a dialect spoken by members of the Indigenous Mexican Purépecha from Michoacan Mexico. Most patients reside near and work in the surrounding agricultural fields. Employment in this region is primarily seasonal agricultural labor and is often characterized by low-wage and economically precarious work [9, 15].

### Participant eligibility

Participants were eligible if they had accessed primary care services at the free primary care clinic, had screened positive for depression (score of 5 or higher on the PHQ-9) [13] or anxiety (score of 5 or higher on the GAD-7) [16] per the clinic’s screening process, and accessed same-day co-located mental healthcare services at the clinic. A CHW at the free clinic shared a study flyer with all patients who met this criteria and invited them to join the study. The clinic accepts patients with or without health insurance and services are free of cost reducing barriers to healthcare access in the patient population. Interested patients either enrolled at the clinic via the CHW. Although gender was not an inclusion criterion, all participants who enrolled during the study period identified as women. All participants (*N* = 12) identified as either Hispanic or Latino women and had a mean age of 46.5 years (SD = 12.17). The majority (*n* = 11) were born in Latin America, predominantly Mexico, and spoke Spanish as their primary language; 11 had completed high school or had less education, and 8 were employed as farmworkers. Four earned less than $25,000 annually, 5 reported a history of unstable housing, and eight lacked health insurance. Most participants lived in households with five or more people (*n* = 7), and nine had children under 18. During the pandemic, nine tested positive for COVID-19, and all received the vaccine. Despite pronounced barriers, 11 had previously accessed mental health services at least once, primarily in the form of therapy.

### Data collection

Qualitative, semi-structured interviews were conducted via Zoom or phone. Interviews lasted 45 to 60 min and explored participants’ experiences with trauma, mental health, and access to mental health resources during the COVID-19 pandemic.

Trained interviewers used a semi-structured interview guide with open-ended questions on three key areas: the impact of the pandemic on mental health, including: experiences of anxiety, depression, and grief; barriers to accessing mental health services, economic instability, linguistic isolation, and cultural stigma; and the role of cultural and social factors, including family dynamics and community support networks (see Supplementary File for interview guide). Prior to the interview, a CHW administered a brief socio-demographic survey collecting age, gender, employment, education, household income, previous use of mental healthcare services, and COVID-19 testing and vaccination history. Participants received a $25 gift card for their time.

### Data analysis

Qualitative data were analyzed using a blended deductive and inductive approach informed by principles of grounded theory described by Strauss and Corbin [17, 18]. Transcripts were uploaded to MAXQDA for systematic coding and data management [19]. As an initial step, we used open coding, a line-by-line analysis of the text, to generate preliminary concepts emerging from the data. During this stage, in vivo codes based on participants’ language were used when phrases captured key ideas and experiences. Following initial open coding, we used a collaborative approach to develop a codebook that defined each code and its application to textual data [20]. The codebook was applied across transcripts and iteratively refined to ensure rigor and consistency in code application. Through axial coding, related codes were grouped into broader analytic categories and relationships between categories were identified, permitting a comparative analysis of patterns across participants’ experiences.

Regular discussions among researchers facilitated iterative refinement of the analysis. Through these discussions, we recognized that the emerging analytic categories reflected multiple levels of influence, consistent with the Social Ecological Model (SEM) [11]. This prompted further deductive analysis using the SEM as an organizing framework to examine how systemic, macro-level societal factors (e.g., economic structures, government policies) and meso-level factors (e.g., social networks, community environments, public health and healthcare system resources and infrastructure) intersect with and shape individual-level mental health outcomes. As such, analytic categories were examined in relation to these multiple spheres of influence to identify how systemic and contextual factors intersected with individual experiences to shape mental health outcomes both before and during the COVID-19 pandemic.

## Results

**Fig. 1:**
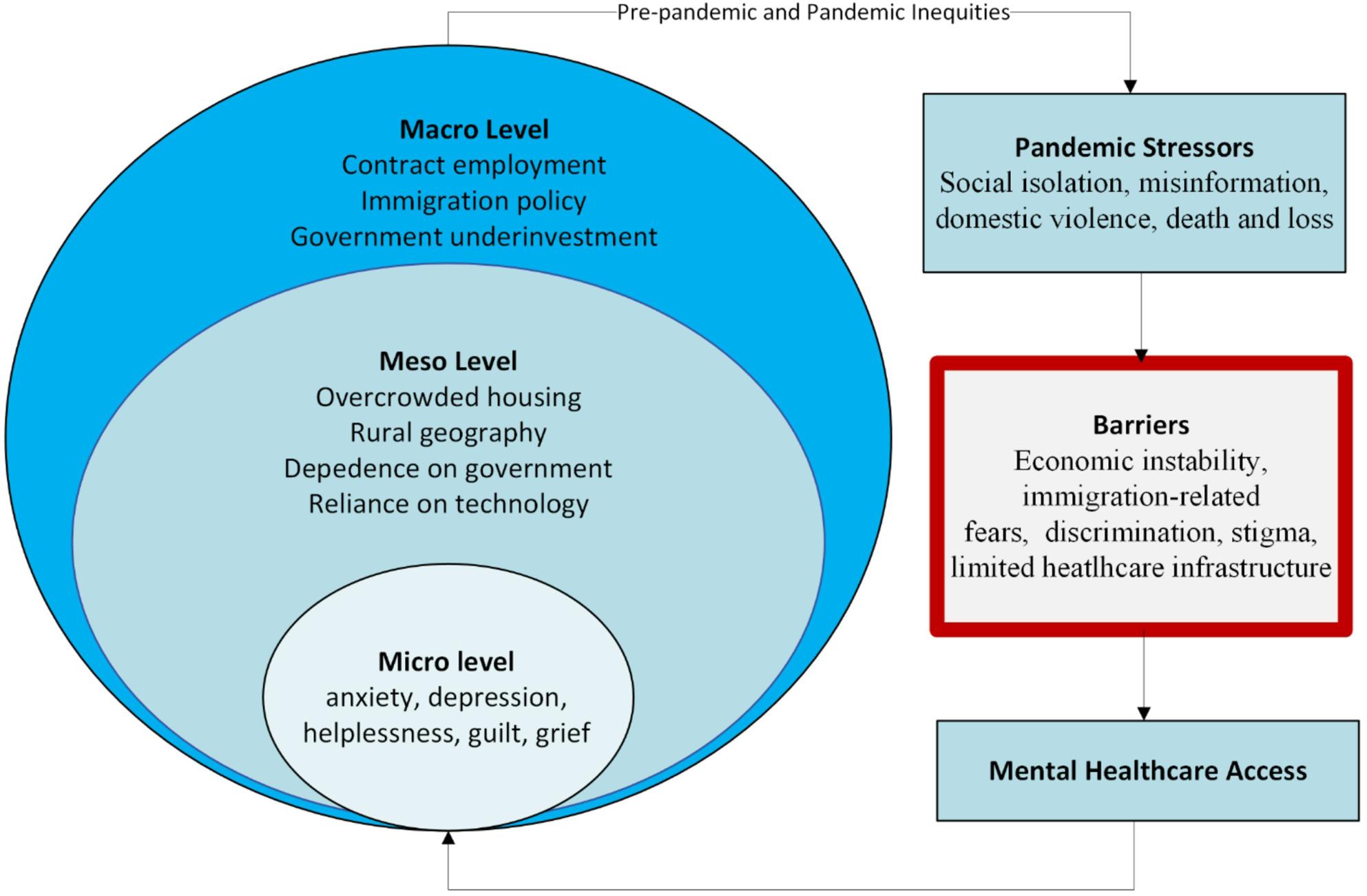
Contextualization of participants’ mental health experiences within the SEM

Figure 1 situates participants experiences within the SEM, illustrating how structural (macro-level), community, interpersonal, and individual factors intersected to shape mental health outcomes and help-seeking decisions in rural Latinx and Indigenous Mexican farm-working communities. Participants described experiences of uncertainty, helplessness, and guilt as emerging from structural and social inequities that were exacerbated during the pandemic. Structural inequities across multiple spheres of influence made some individuals more vulnerable to the effects of such inequities on mental health outcomes resulting in psychological and emotional distress. This occurred in a context of limited healthcare infrastructure. Below, we discuss how structural and social conditions shaped participants’ mental health experiences during the pandemic and challenges to accessing mental health care services in a rural geography with limited healthcare infrastructure.

### Structural-level factors: vulnerability to inequities

Participants described long-standing inequities that shaped vulnerability to stress and limited access to healthcare resources and services prior to the pandemic. These included contingent, low-wage labor as agricultural laborers, immigration-related exclusion from health insurance, and underinvestment in healthcare infrastructure.

Employment conditions contributed to chronic daily stressors related to financial strain, necessitating a focus on basic needs such as food and housing over health. Participants described working long hours: “Here, you have to work all the time, you have to get up, and you spend more time at work than at home.” Others shared that, at times, there was not enough work: “There is lack of work and there is not enough for your children.” A different participant echoed this point: “The hard part is, because with the heat we are having this season, there is very little work. Not everyone can work.” For seasonal employees, particularly farm workers, they experienced additional stressors related to migration patterns, harvest seasons, and extreme weather (e.g., heat). “Those who work the fields suffer the most,” a participant shared.

Racialized immigration policy was a pervasive structural condition that excluded undocumented individuals from government-funded insurance (i.e., Medi-Cal) and generated fear of deportation when accessing healthcare. As many shared, fear of having to disclose documentation status was a significant deterrent to healthcare services use. “The first thing they ask you is, ‘Do you have insurance?’ And sometimes many people don’t have it because of a lack of documents” a participant commented. Consequently, many forgo needed services: Anti-immigrant rhetoric and policy contributed to discriminatory experiences, particularly within healthcare settings. A participant commented:

We are a community, that experiences racism. You go to the hospital, and just because of where you’re from [rural farm-working community], that’s enough for them to treat you differently. They speak to you differently, they look at you differently.

Underinvestment in healthcare exacerbated these challenges. Participants described a lack of healthcare providers, especially mental healthcare providers, as well as limited availability of culturally and linguistically appropriate services. For instance, this participant discussed the limited services and providers: “We need more services because they are very limited. The ones available nearby hardly ever allow you to see someone in person, like a psychologist or psychiatrist.” Echoing this point, a different participant commented: “I do have the right to the service, and the insurance does cover it, but we don’t have specialists. They haven’t hired any.”

### Community-level factors: pandemic disruptions and collective experience

Against this backdrop of existing inequities, the pandemic introduced new pressures that intensified distress across households and the broader community. The COVID-19 pandemic limited in-person interactions, reducing support systems, and shifting to online education and digital communication. Isolation limited contact with relatives, neighbors, and support networks. Participants described how the stress and frustration of confinement and social isolation was compounded by overcrowded housing limiting privacy and personal space. As one participant explained, “Most live with more than two families in a small apartment or small house, and sometimes up to three or four families.”

Job loss and financial insecurity contributed to hardship for families and communities pushing many to ask for rental and utility assistance. A participant shared: “A whole line of people on the benches there, people asking for rental assistance and help with utility bills. And who were they? All my neighbors.” Some experienced significant hardships—as this participant commented: “There are a lot of people who need help because many lost their jobs and some lost their houses.”

Virus spread, illness, death, and misinformation had profound community impacts. Participants described the loss of family members, neighbors, and trusted community leaders, including religious figures, whose death deeply affected collective systems of emotional and spiritual support. A participated reflected on the death of the local catholic priest: “We had a priest who passed away due to COVID. A large part of our community sought resources and support because we were deeply saddened; we lost someone we would see there every Sunday.” The inability to grieve and mourn with others and in religious settings due to pandemic restrictions contributed to unresolved grief and collective and personal loss.

### Interpersonal-level factors: household dynamics and relational strain

Participants described pandemic stressors as intensifying dynamics within households, particularly in overcrowded living conditions experiencing economic strain. Confinement, in combination with unemployment and pandemic-related uncertainty, contributed to tension, conflict, and, in some cases, domestic violence. Participants experienced disruptions to daily life and routines, which limited privacy and increased the frequency with which household members interacted, creating stress within family and interpersonal relationships. A participant shared:

Having to be confined inside with the kids. It’s sometimes challenging to live in the same space as your own partner and family . . . we are stuck inside . . . we hear about people having terrible anxiety.

These type of disruptions were especially impactful in households with existing conflict, heightening vulnerability to domestic or interpersonal violence. Participants described violence against women and children as being “common in our community” yet more commonplace during the pandemic. A participant described the escalation of violence within her home: “My husband became more aggressive when the pandemic started. . he couldn’t go out, he couldn’t do anything, and he took it out on us.” Others comments on seeing or hearing violence in their community. Narrating observations of her neighbors, this participant shared: “Many times, if the mother is on the phone and the father is anxious, stressed because there is no work, conflicts arise—they start yelling [at each other].”

Participants also talked about changing roles and responsibilities within families as parents and caregivers had to help children navigate remote, online schooling. Many experienced challenges with limited broadband connectivity and unfamiliar digital platforms, which created stress and strained parent-child relationships. A participant shared the following experience: “Losing the [internet] signal every so often, and my daughter not finishing (school). And [feeling] this hopelessness for not being able to help with it. It was very stressful.”

Concern over children’s wellbeing, given their limited interaction with peers, struggles with online learning, and fear of virus spread, was prominent in participants’ narratives. Several described children expressing fears about illness, death, and the possible loss of loved ones, whereas parents experienced stress having to protect their children and families from instability and uncertainly due to pandemic stressors. During the interview, a participant reflected on her daughter’s fears: “my daughter would say, ‘What if my baby dies, mom?’”.

### Individual-level factors: embodiment of psychological distress

Across the interviews, participants described experiencing anxiety, depression, insomnia, and grief as responses to prolonged uncertainty, loss, and social constraint. Uncertainty was at the crux of psychological distress. This included uncertainty about virus spread, as this quote illustrates: “When there is a sick person (at home), it’s stressful. . . . or if you don't know [you have COVID-19] . . . then you could end up transmitting it to your family.” It also included uncertainty about their current situation and the pandemic more generally. Reflecting on her family’s experience, this participant shared: “We felt alone. It was harder to accept a situation we didn’t understand.” Other’s talked about insomnia and how they or members of their household “can’t sleep due to worry.”

Feelings of helplessness and guilt were also common. Participants described frustration with insufficient resources, not having enough money to buy food, limited support for their children’s education, and needing to protect themselves and their family from illness. Some expressed the guilt related to getting COVID-19 and spreading it throughout their households, reflecting the emotional toil of navigating risk within constrained and overburdened households. A participant shared: “I was the one who got it the worst, with the cough and all that, one of my daughters who brought COVID home felt guilty.”

Grief was another common experience. Many participants lost family members, neighbors, and esteemed community leaders (e.g., priest) to COVID-19. Due to social distancing, participants were unable to grieve with others to mourn the loss of loved ones. Some discussed unresolved grief and struggling to process repeated losses amidst providing for basic and daily individual and family needs. “I lost my father to COVID. . . I still haven’t been able to overcome that,” shared a participant. Reflecting on their loved one’s experience, this participant commented: “Since many people close to us passed away . . . that’s when she [loved one], began to feel panic and fear.” Several noted the enduring impact of the pandemic: “The people who lost loved ones due to COVID—this pandemic will leave a mark for many years to come.”

### Barriers to mental healthcare

Inequities embedded within the multiple levels of the SEM created barriers to healthcare access. At the structural level, economic insecurity and lack of insurance limited the affordability of services. A participant commented: “I did not have money to even eat. How would I search for help with my mental health?” Immigration-related fears discouraged engagement with healthcare systems, particularly when documentation was required. One participant shared: “I knew I needed help, but without papers, I couldn’t go anywhere. I was afraid I would be detained.” Furthermore, participants described heightened experiences of discrimination in healthcare settings. “With everything we are going through with COVID—it’s really difficult because the clinics we have here, in the area, don’t want to care for you,” a participant shared.

At the community level, limited availability of local services and lack of culturally and linguistically appropriate care reduced accessibility to mental health services. Cultural stigma further shaped decisions about help seeking. Participants described mental health struggles as hidden or taboo topics within their communities. As one participant explained, “In our community, we don’t talk about those kinds of things—it’s taboo.” Another participant similarly shared:

We are ashamed to talk about problems. . . because we think they will judge or criticize us, or wonder what they will say [to others]. And, these are topics that, in our culture, it’s like: ‘That has to stay hidden. You have to keep that to yourself.

At the interpersonal level, stigma and concerns about privacy within close-knit communities discouraged opening up about mental health challenges and care seeking discussions. Participants feared being judged or the social repercussions of disclosing mental health struggles, leading many to conceal their distress rather than seek support.

At the individual level, these factors shaped perceptions of care as inaccessible, unsafe, or unnecessary. Participants often prioritized immediate survival needs such as food and housing over mental healthcare, particularly in the context of financial strain.

As participants’ narrative illustrate, the pandemic intensified existing inequities across the levels of the SEM, structural, community, and interpersonal, which both contributed to individual level psychological and emotional distress and constrained pathways to help seeking and mental healthcare service use. “Out of fear of not having papers, people endure a lot of things,” a participant remarked.

## Discussion

This study highlights the systemic inequities that shaped mental health outcomes for low-income Hispanic/Latino immigrants in rural farm working communities in Inland Southern California. The contextualization of our findings in the multiple spheres of influence defined by the socioecological framework [11] highlights how the intersection of pre-pandemic and pandemic conditions at the macro-level with the meso-level exacerbated existing inequities and stressors that played out at the individual level contributing to poor mental health outcomes during the pandemic.

These findings underscore how structural inequities uniquely manifest in rural desert farm working communities such as the Eastern Coachella Valley, where geographic isolation, immigration-related fear, linguistic barriers, and under-resourced safety-net systems converge to shape mental health outcomes [12, 21]. Unlike urban settings with denser healthcare infrastructure, participants described reliance on the free clinic and CHWs as their primary point of access to mental health support [12, 22], amplifying the consequences of service gaps, mistrust, and delayed care. Situating these findings in this local context highlights the importance of culturally and linguistically responsive mental health services that address the structural conditions that shape healthcare access for rural immigrant communities [23, 24].

Our research and that of others shows how the COVID-19 pandemic widened existing inequities, disproportionately affecting low-income Latinx and Indigenous populations, who faced higher morbidity, mortality, and limited access to mental health resources [1, 3, 25]. Pre-pandemic conditions at a systems level, including governmental underinvestment, economic instability, and structural racism, contributed to overcrowded housing, precarious employment, and restricted healthcare access in historically disadvantaged populations creating the conditions for increased virus exposure and COVID-19 transmission [12, 21].

These inequities stem from historical exclusion, exploitation, and discriminatory immigration policies, further intensified by polarized political discourse and fluctuating federal policies. This climate fostered distrust in government institutions, discouraging participation in healthcare programs as a perceived safety measure [26, 27]. Among immigrant populations, the fear of deportation compounded these challenges. In our study, such fear prompted participants to prioritize family safety over individual mental health care, even amid severe anxiety and depression. This aligns with research on the psychological impacts of immigration enforcement, where fear deters essential healthcare engagement [28].

The living and working conditions of historically disadvantaged populations, such as overcrowded housing and low-wage contingent labor, became untenable during the pandemic, underlying domestic violence and strained familial relationships. Kourti et al.’s systematic review of domestic violence global trends during the COVID-19 pandemic reveals increased rates of exposure, especially during the early days of the pandemic [29]. In minority communities, COVID-19 public health mitigation efforts of social distancing disrupted traditional community networks that typically buffer against stress [30]. Furthermore, widespread misinformation and perceived inadequacies in public health responses deepened distrust in healthcare systems and public resources intensifying feelings of helplessness, guilt, and grief and the need for community networks [25, 31, 32].

### Public health implications

These findings have specific implications for rural desert farm working communities in the Eastern Coachella Valley, where geographic isolation, seasonal agricultural labor, immigration-related fear, and reliance on a limited number of safety net clinics shape access to mental health care [1, 12]. Anchoring these implications in this local context clarifies how structural and community-level conditions translated into individual mental health burden during the COVID-19 pandemic [1].

At the macro level, these findings point to structural policy interventions that address the upstream conditions shaping mental health access in rural desert farm working communities. Participants’ accounts of overcrowded housing, confinement-related stress, and lack of privacy highlight the need for housing policies that mitigate residential crowding in farmworker communities, consistent with evidence linking housing insecurity to adverse mental health outcomes during the COVID-19 pandemic [33]. Economic precarity driven by contingent and seasonal agricultural labor underscores the importance of labor protections and income stability measures to reduce pandemic-related stress and mental health strain [19, 34]. In addition, fear of deportation and exclusion from formal systems indicate a need for immigration-safe access to publicly funded mental health and social services, aligning with prior research demonstrating how immigration enforcement climates deter healthcare utilization and exacerbate psychological distress [35].

Although participants were able to access services through a safety-net clinic that provides care regardless of insurance or immigration status, lack of health insurance limited access to specialty care, continuity of treatment, and services beyond the limited scope of the clinic. Furthermore, the clinic provides services once a month and appointments are limited as only one therapist is on staff per clinic. Participants described delays, lack of specialists, and referral barriers indicating that insurance coverage remains structurally relevant to sustained mental health care access.

At the meso-level, community-based strategies must focus on rebuilding trust and strengthening social networks that buffer against stress [23]. Partnerships with CHWs can provide an essential bridge between healthcare and public health systems and communities, facilitating access to care and fostering trust [22]. In this study, CHWs were trusted community members who supported outreach, screening, navigation, and culturally responsive communication, illustrating the skill set required to effectively address structural and cultural and linguistic barriers. Culturally relevant health education, designed with community input, that reduces stigma and empowers individuals to seek care is also crucial [24]. Strengthening community networks offers a vital counterbalance to social isolation and provides individuals with supportive environments to navigate systemic barriers and address their mental health needs [23].

### Limitations

This study has several limitations. The sample size was small and drawn from patients who accessed co-located mental health services at a single free clinic offering once monthly services only, which may limit generalizability to other rural immigrant communities or to individuals who did not seek care. All participants identified as women, which precluded a comparative gender-based analysis and may not capture the experiences of men or individuals of diverse gender identities. That said, the all-women sample highlights the gendered nature of help-seeking, especially mental health care, within Latino communities where values of familism and gender-role expectations position women as primarily responsible for family and community health [36]. Such gender expectations may make women more likely to recognize mental health needs and possess knowledge of healthcare options. Another limitation is the findings are based on retrospective self-report of pandemic experiences and may be subject to recall bias. The qualitative design prioritizes depth of understanding over representativeness, and results should be interpreted accordingly.

### Future directions

An important contribution of this study is its demonstration of how structural inequities shape mental health care access in rural immigrant communities. Findings show that geographic isolation, immigration-related fear, language discordance, and limited safety-net infrastructure contribute to both daily stressors and barriers to care [9]. This study further illustrates how inequities embedded within social and institutional contexts, including political, economic, and healthcare systems, influence who can and cannot access services. These findings underscore the need for future research to explicitly consider systemic inequities experienced by structurally vulnerable populations and to incorporate these realities into the design of intervention models that address barriers within healthcare delivery systems while aligning with the cultural and linguistic needs of diverse patient populations [37–39].

Future research should also include men and individuals of diverse gender identities to examine whether mental health experiences and access barriers differ by gender in rural immigrant communities. Studies that engage community members who did not access mental health services during the pandemic are needed to better characterize the unmet need and persistent barriers to care. Longitudinal research examining mental health trajectories beyond the acute pandemic period would help clarify the durability of anxiety, depression, and grief described in this study. Additional work evaluating community health worker-led and language-concordant mental health models could assess their effectiveness in improving access and engagement in rural desert regions.

## Conclusion

Our study shows how the pandemic conditions perpetuated a cycle of unmet mental health needs, exacerbating existing social, economic, and political inequities and creating unique pandemic stressors heightening anxiety, depression, and guilt within rural Hispanic/Latinx immigrant communities. By applying a socioecological framework, this study provides critical insights into the interplay between structural and social inequities and individual mental health outcomes, offering a foundation for future research and interventions. Addressing these disparities requires structurally and culturally tailored, solutions that reduce inequities and rebuild trust and improve access to care in underserved populations [40]. Persistent barriers such as linguistic isolation, fear of deportation, and stigma must be addressed to break the cycle of unmet mental health needs [12], and access to bilingual and bicultural healthcare providers are critical steps [12, 24].

## Supplementary Information

Supplementary Material 1.

## Data Availability

The datasets used and/or analyzed during the current study are available from the corresponding author on reasonable request.
