# Supplementary material for: Mental health care access among Latinx and Indigenous Mexican immigrants in rural desert communities in the COVID-19 pandemic: a community-engaged study
Source: BMC Public Health. 2026 May 4;26:1929. doi: 10.1186/s12889-026-27551-6 (PMC13285207; doi:10.1186/s12889-026-27551-6)
Supplement: Supplementary file 1 — Supplementary Material 1. [file 12889_2026_27551_MOESM1_ESM.pdf]

Supplementary File: Qualitative Interview Guide

Introduction: Thank you for taking the time to meet with me today. We asked you to do this interview so we can get a better understanding of use of behavioral healthcare services this includes mental health and substance use services in your community. First, we'll ask you to talk about the free clinic and then about the kinds of behavioral healthcare services in your community. We will also ask you to share your thoughts about some challenges to getting care for mental health and substance use and what would make it easier, especially during the COVID-19 pandemic. Remember there are no right or wrong answers. We would like to record this interview. While audio recording is not required for participation, it is preferred. If participants do not want to be audio recorded, notes will be taken to document information shared. Do we have your permission to record the interview?

Questions:

1. Can you tell me about the kinds of stress and trauma that people in your community have experienced during the COVID-19 pandemic?
   a. Probe: Would you like to share with me any example of stress or trauma that you or someone in your family has experienced?
2. Now let's talk about your visit to the clinic. Can you tell me about why you decided to go to the Coachella Valley Free Clinic? What brought you to the clinic?
   a. Probe:
      i. How did you hear about the clinic?
      ii. What did you learn about our mental health services?
      iii. How did you get to the clinic? (car, bus, walked)
      iv. Did you experience any challenges to getting to the clinic?
3. What did you think of your visit with the mental healthcare professional?
   a. Probe:
      i. Was it helpful or not? Why?
      ii. What about where the visit was held, were you comfortable, uncomfortable in the space? Was there enough privacy, why or why not? (probe around stigma)
4. Now let's talk about behavioral health care services available in your community. What kind of services already exist in your community?
   a. Probe:
      i. Services through the church or other organizations?
      ii. Services for individuals and families that help with trauma/stress related to the COVID-19 pandemic (e.g., grief, death, job loss, child anxiety)
      iii. Have you ever used these services, why or why not?

5. In your opinion, how could this clinic better address mental health and substance use needs in your community?

6. Is there anything else you'd like to share related to your experiences with using the services available at this clinic?

Guía de entrevista cualitativa

<u>Introducción:</u> Gracias por tomarse el tiempo para reunirse conmigo hoy. Le pedimos que hiciera esta entrevista para que podamos entender mejor el uso de los servicios de salud conductual, esto incluye servicios de salud mental y el uso de sustancias en su comunidad. Primero, le pediremos que hable sobre su experiencia en la clínica gratuita y luego sobre los tipos de servicios de atención médica conductual que existen en su comunidad. También, le pediremos que comparta sus pensamientos sobre algunos desafíos para obtener atención de salud mental y el uso de sustancias, y lo que facilita el acceso a estos recursos, especialmente durante la pandemia de COVID-19. Recuerda que no hay respuestas correctas o incorrectas.
Nos gustaría grabar esta entrevista. Si bien no se requiere grabación de audio para la participación, pero se prefiere. Si usted no desea que grabemos el audio, se tomarán notas para documentar la información compartida. ¿Tenemos su permiso para grabar la entrevista?

<u>Preguntas:</u>

1. ¿Puede contarme sobre los tipos de estrés y traumas que las personas en su comunidad han experimentado durante la pandemia de COVID-19?

    Indague: ¿Le gustaría compartir algún ejemplo sobre estrés o trauma que usted o alguien de su familia haya experimentado?

2. Ahora hablemos de su visita a la clínica. ¿Puede contarme por qué decidió ir a la Clínica Gratuita del Este del Valle de Coachella? ¿Por qué razón visitó la clínica?

    Indague:

    - ¿Cómo se enteró de la clínica?
    - ¿Qué aprendió de nuestros servicios de salud mental?
    - ¿Cómo llegó usted a la clínica? (por carro, autobús, caminar)
    - ¿Tuvo alguna dificultad para llegar a la clínica?

3. ¿Qué le pareció su consulta con el profesional de la salud mental?

    Indague:

    - ¿Le fue útil, o no? ¿Por qué?
    - ¿Qué pasa con el lugar donde se llevó a cabo la visita, qué tan cómodo(a) o incómodo(a) en el espacio? ¿Tuvo suficiente privacidad? ¿por qué o por qué no? (preguntar sobre el estigma)

4. Ahora hablemos de los servicios de atención de salud conductual que existen en su comunidad. ¿Qué tipo de servicios de salud mental y del uso de sustancias existen en su comunidad?

    Indague

    - ¿Servicios a través de la iglesia u otras organizaciones?

- Servicios para individuos y familias que ayudan con el trauma / estrés relacionado con la pandemia de COVID-19 (por ejemplo, dolor, muerte, pérdida de trabajo, ansiedad infantil)
- ¿Alguna vez ha utilizado estos servicios, por qué o por qué no?

5. En su opinión, ¿cómo podría esta clínica satisfacer mejor las necesidades de salud mental y uso de sustancias en su comunidad?

6. ¿Hay algo más que le gustaría compartir relacionado con sus experiencias con el uso de los servicios ofrecidos en esta clínica?
